# Local recurrence as extraocular muscle lymphoma after 6 years of chronic myositis: a case report

**DOI:** 10.1186/s12886-022-02623-4

**Published:** 2022-10-08

**Authors:** Qihan Guo, Rui Liu, Xuan Zhang, Bentao Yang, Jianmin Ma

**Affiliations:** 1 Beijing Tongren Eye Center, Beijing Ophthalmology and Visual Science Key Lab, Beijing Tongren Hospital, Capital Medical University, Beijing, China; 2 Ophthalmology, Taian City Central Hospital, Tai’an, Shandong Province China; 3 Radiology, Beijing Tongren Hospital, Capital Medical University, Beijing, China

**Keywords:** Extraocular muscle, Myositis, IgG4-related ophthalmic disease, MALT lymphoma, Case report

## Abstract

**Background:**

Extraocular muscle is usually affected by thyroid disease or inflammatory pseudotumor, but seldom by neoplastic process. Primary malignant lymphoma involving isolated extraocular muscle is very rare, especially after 6 years of chronic myositis.

**Case presentation:**

A middle-aged female presented with swelling of the lower lid of the right eye for 2 months. Magnetic resonance imaging showed significant enlargement of the right inferior rectus muscle belly. The patient first presented 6 years prior with upper eyelid swelling. A total of 5 surgical biopsies of the right eye were performed during 6 years with the following successive findings: inflammatory pseudotumor, chronic inflammation, inflammatory lesions, IgG4-related ophthalmic disease, and lastly, extraocular muscle extranodal marginal zone B-cell lymphoma of mucosa-associated lymphoid tissue (MALT lymphoma).

**Conclusion:**

MALT lymphoma may have occurred as a result of chronic extraocular myositis. Malignancy should be considered in patients with recurrent painless extraocular muscle hypertrophy. Differential diagnosis can rule out thyroid-associated ophthalmopathy (TAO), whose symptoms are similar. Diagnosis confirmation by biopsy is warranted if necessary.

## Background

Isolated extraocular muscle enlargement is typically associated with thyroid disease and idiopathic orbital inflammation, but seldom with neoplastic process. These etiologies may share similar clinical and radiological features, making the correct diagnosis challenging. Primary malignant lymphoma involving isolated extraocular muscle is extremely rare. Here, we describe the clinical, radiological, and pathological features of a biopsy-confirmed mucosa-associated lymphoid tissue (MALT) lymphoma of the inferior rectus muscle after 6 years of myositis. We include an analysis of a possible association between the lymphoma and IgG4-related inflammation.

## Case description

**Fig. 1:**
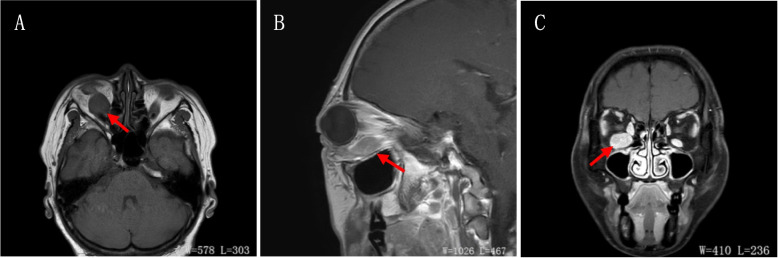
Magnetic resonance imaging of the right inferior rectus muscle enlargement (red arrow) in 3 different images: **A** T1-weighted axial image; **B** T1-weighted sagittal image; **C** Post-contrast T1-weighted coronal image

A 55-year-old female presented with swelling of the lower lid of the right eye for 2 months without redness, ocular pain, or decreased visual acuity. This patient had a long and complicated course for 6 years, as shown in Table 1. Ophthalmic examination indicated a visual acuity of 20/20 in the right eye and 20/25 in the left eye and intraocular pressure of 12 mmHg bilaterally. The right eye showed proptosis, lower lid swelling, mild conjunctival injection, and limited ocular motility when looking upward. Other aspects of the examination, including the anterior segment, optic disc, and fundus, were within normal limits in both eyes. There was no palpable regional lymphadenopathy. Magnetic resonance imaging showed significant enlargement of the right inferior rectus muscle belly (Fig. 1). Laboratory tests showed normal complete blood count, hyperuricemia, hyperlipidemia, elevated serum complement factor 4 (49.5 mg/dl), and elevated C-reactive protein (6.2 mg/L). Thyroid function tests were normal, including serum total T3, total T4, free T3, free T4, thyroid-stimulating hormone, thyroid stimulating hormone-receptor antibodies, thyroglobulin antibody, and thyroid microsomal antibody.

**Table 1:** Records of five visits within 6 years

Time	Age	Clinical presentation	Anatomical location	Laboratory findings	Pathology
2015.10	49	swelling of the upper eyelids of both eyes	lacrimal gland in both eyes right inferior rectus muscle	serum IgG4: 132.0mg/dl thyroid function test (-) autoimmune serology (-) EBV antibody(-)	right lacrimal gland: inflammatory pseudotumor left lacrimal gland: IgG4-ROD
2019.01	52	proptosis and swelling of the lower lid of the right eye	right inferior rectus muscle	thyroid function test (-)	chronic inflammation in striated muscles tissue with lymphoid follicle formation
2019.10	53	swelling of the lower lid of the right eye	right inferior rectus muscle	thyroid function test (-)	focal lymphocytic proliferation with scattered plasma cells, follicle formation, a few collagen deposits
2020.11	54	proptosis and swelling of the lower lid of the right eye	right inferior rectus muscle	thyroid function test (-)	diffuse lymphocytic proliferation with massive plasma cells infiltration in certain areas, follicle formation, mesenchymal fibrosis; IgG4-ROD
2021.10	55	swelling of the lower lid of the right eye	right inferior rectus muscle	thyroid function test (-)	extranodal marginal zone B-cell lymphoma of mucosa-associated lymphoid tissue

*EBV* Epstein–Barr virus, *IgG4-ROD* IgG4-related ophthalmic disease

**Fig. 2:**
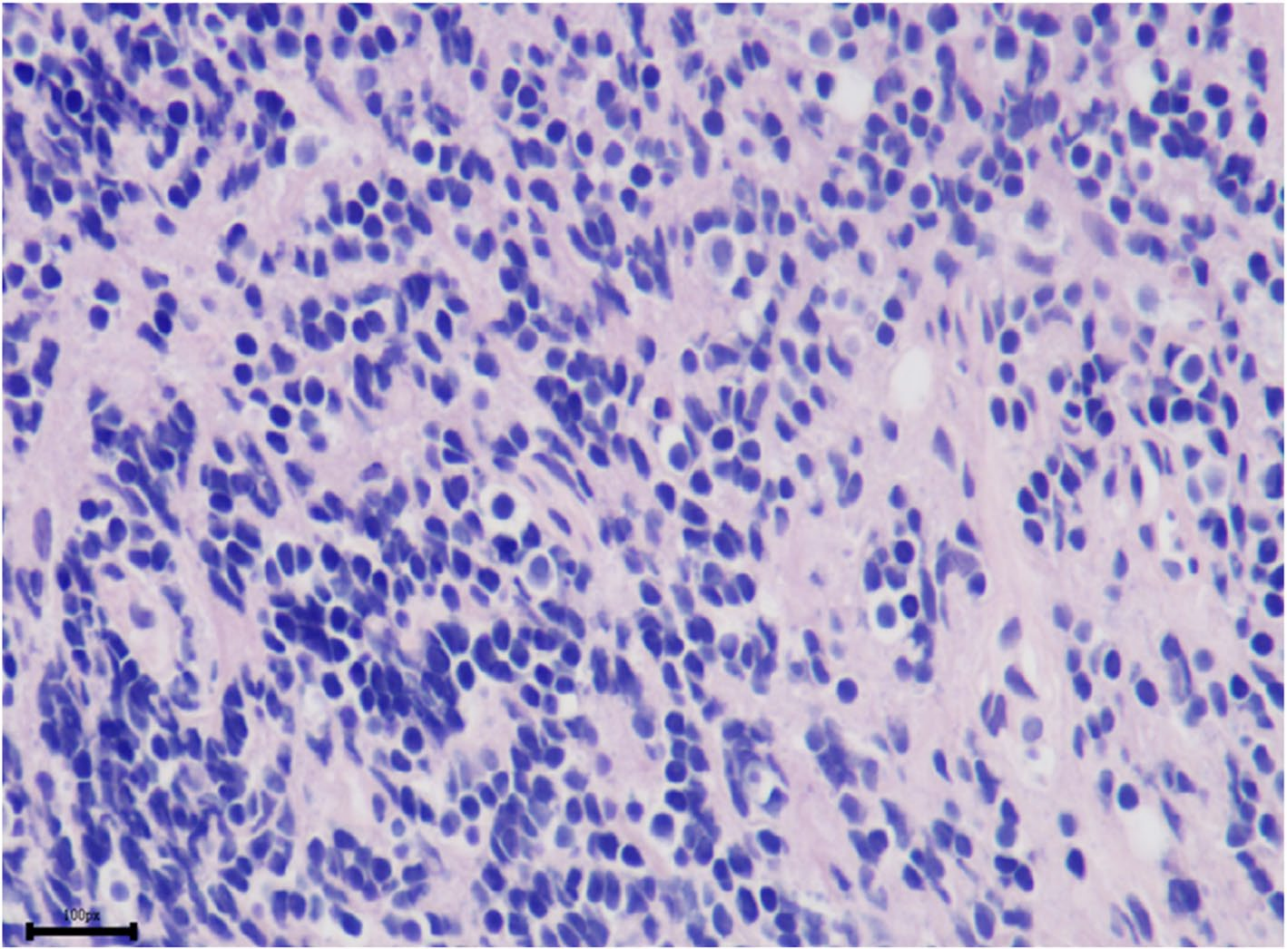
Massive lymphocytic proliferation with slight nuclear atypia. (H&E stain: × 400, resolution: 781 × 628 pixel)

**Fig. 3:**
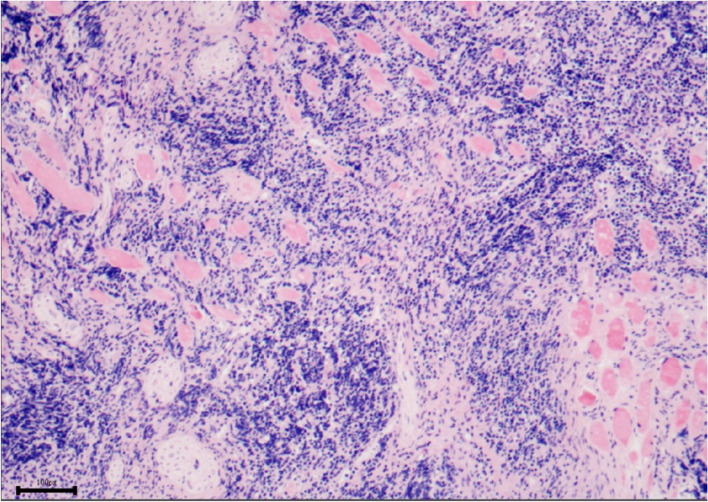
Invasion of tumor cells in striated muscle tissue. (H&E stain: × 100, resolution: 1076 × 762 pixel)

**Fig. 4:**
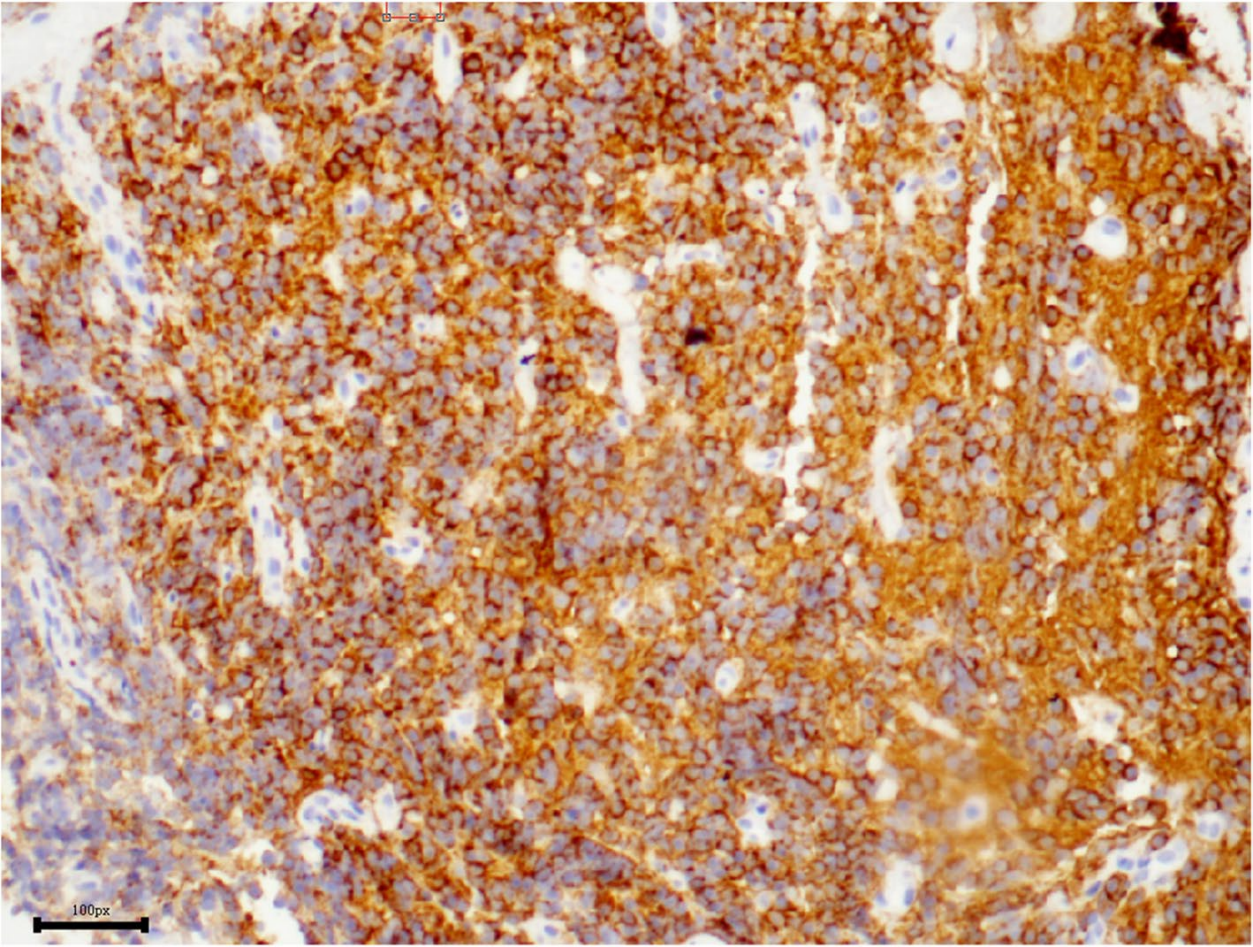
Immunohistochemical profile of the lymphocytic proliferation showing expression of cells to CD20. (× 200, resolution: 1116 × 843 pixel)

**Fig. 5:**
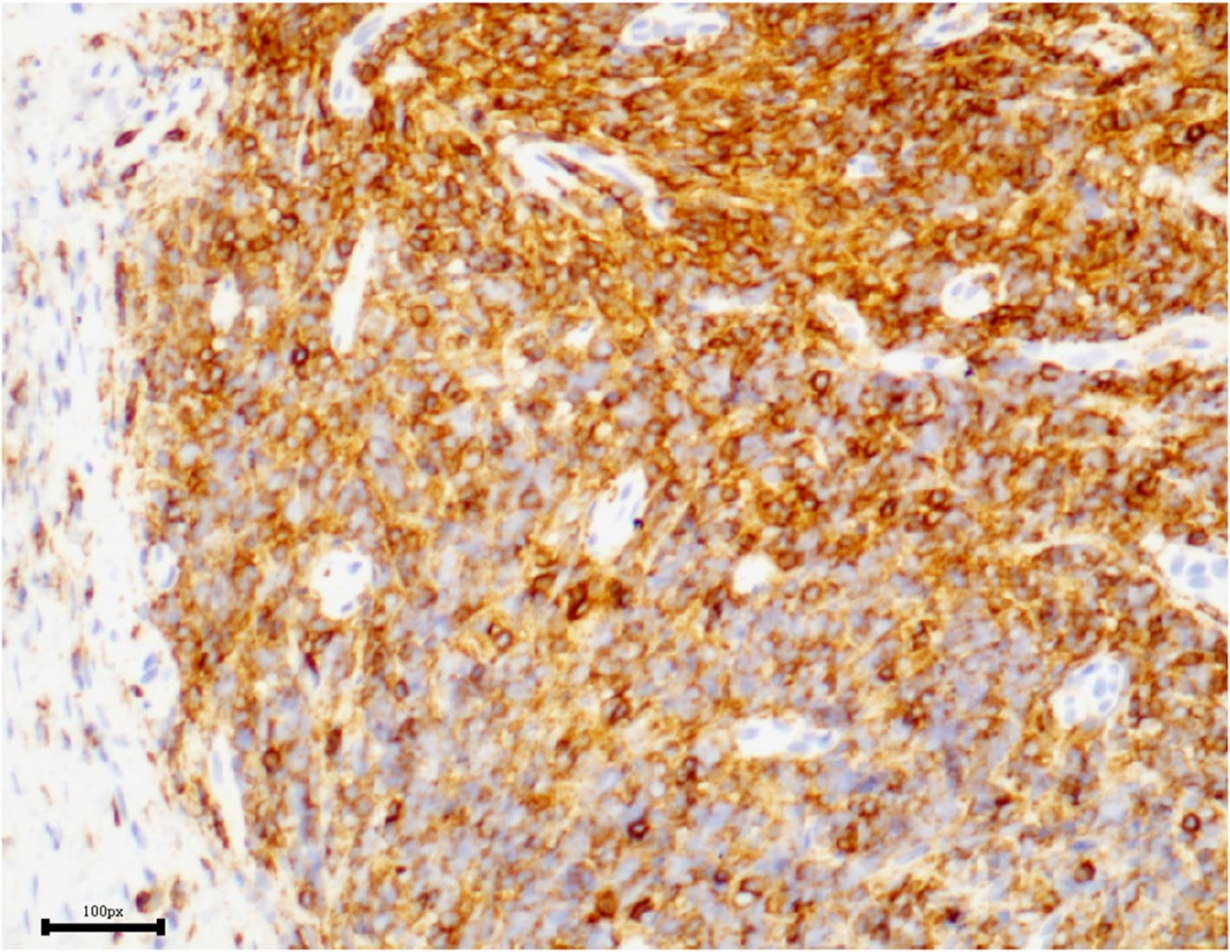
Immunohistochemical profile of the lymphocytic proliferation showing expression of cells to CD43. (× 200, resolution: 1020 × 789 pixel)

**Fig. 6:**
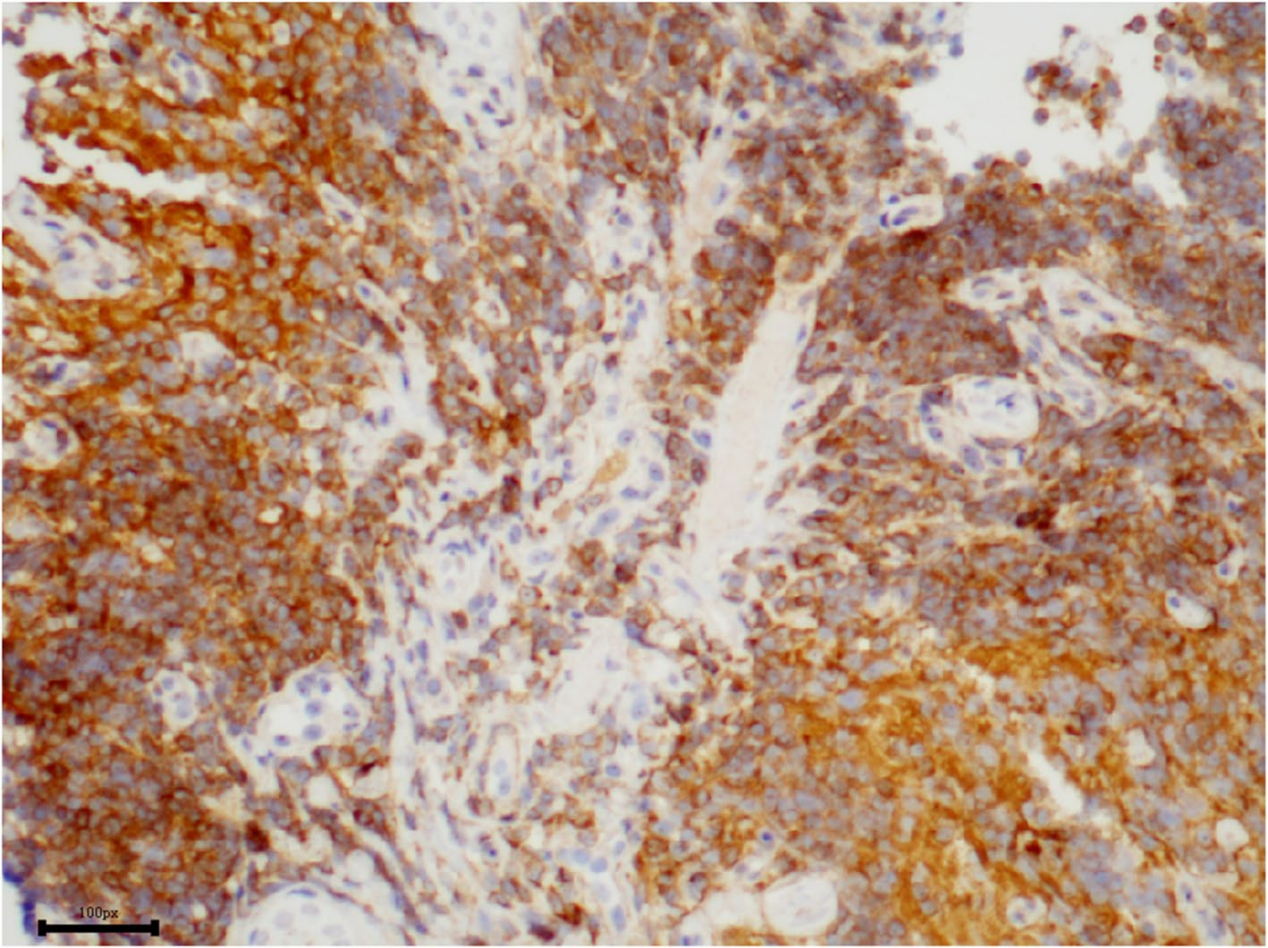
Immunohistochemical profile of the lymphocytic proliferation showing expression of cells to BCL-2. (× 200, resolution: 1075 × 805 pixel)

Incisional biopsy under general anesthesia was performed. The enlarged right inferior rectus muscle appeared friable with a greyish cut surface. A biopsy was taken from the muscle belly and the hematoxylin-eosin staining of the lesion showed diffuse and dense infiltration by small lymphoid cells with slight nuclear atypia (Fig. 2). In some regions, tumor cells were seen to invade the striated muscle tissue (Fig. 3). Immunohistochemical staining was positive for CD20 (Fig. 4), CD3, CD43 (Fig. 5), CD21 (FDC+), CD138, CD38, SOX10 and Bcl-2 (Fig. 6), and negative for cyclinD1, CD5, and CD10, with a low-level Ki-67 expression of 10%. All pathological specimens were reviewed by a single pathologist. Histologic sections were visualized under an Nikon ECLIPSE Ni-U microscope. The acquisition software is OPLENIC CAMERA.

After the operation, the patient was treated with oral methylprednisolone with an initial dosage of 24 mg per day for 1 week and a taper over 6 weeks. Besides, it was recommended that the patient take radiation therapy in a general hospital. The patient was followed up after 4 months and reported she did not take radiotherapy and had a recurrence of proptosis of the right eye 2 months after the operation. Additionally, the patient had elevated IgG4 levels (124.6 mg/L) in another hospital 1 month after the MALT lymphoma diagnosis. A whole-body PET/CT scan post 18F-FDG administration was conducted and revealed a hypermetabolic retro-orbital mass in the right orbit, considered to be residual tumor after resection, and slightly increased uptake in bilateral submandibular lymph nodes, considered to be inflammation. No abnormally hypermetabolic lymph nodes were seen systemically.

## Discussion and conclusions

Malignant lymphoma is the most common malignancy in the ocular adnexa, and MALT lymphoma has the highest incidence [1]. The orbit is the most common primary site of ocular adnexal MALT lymphoma (46.3–62%), with different studies reporting the conjunctiva (23.4–38%) or lacrimal glands (17.2–34.7%) as the next most common site, followed by eyelids (8.3–18.8%) [2–4]. In recent large cohort studies, no case of ocular adnexal MALT lymphoma involved the extraocular muscles primarily [3–8]. There have been a few isolated case reports in the literature about biopsy-proven ocular adnexal lymphoma with discrete involvement of the extraocular muscles [9–11]. Studies have listed conjunctival injection, pain, and proptosis as the main symptoms. Other reported signs include exotropia and motility limitation, which may suggest impairment of the ocular muscles. Watkins et al. found that the rectus muscles were involved more frequently (11 of 12 cases) than the obliques [12].

Here, we describe a case of primary MALT lymphoma involving the inferior rectus muscle after 6 years of chronic myositis. During that time, the patient underwent 5 incisions in the right eye with the following successive histopathological findings: inflammatory pseudotumor, chronic inflammation, inflammatory lesion, IgG4-related ophthalmic disease, and MALT lymphoma. A progressive increase in the inflammation was noticeable: lymphocytic proliferation evolved from focal to diffuse infiltration, plasma cells increased from scattered to massive infiltration in certain areas, and lymphoid follicle formation occurred throughout the course of the disease. Mesenchymal hyperplasia also gradually intensified, evolving from a small amount of collagenous tissue to mesenchymal fibrosis. The findings at the patient’s first and fourth visits indicate to us that she met the 2014 diagnostic criteria for probable IgG4-related ophthalmic disease [13].

IgG4-related disease (IgG4-RD) is an immune-mediated disease characterized by infiltration of IgG4-immunopositive plasmacytes accompanied by mass-forming lesions in one or more organs. At the first visit of the patient in our case report, serum IgG4 was mildly elevated (132.0 mg/dl) and immunohistological findings showed the ratio of IgG4+/IgG+ plasma cells as 40%, with more than 50 IgG4+ plasma cells/HPF. At the fourth visit, the patient refused to take serum IgG4 examination due to the high price. Immunohistological findings showed the ratio of IgG4+/IgG+ plasma cells as 30%, with more than 50 IgG4+ plasma cells/HPF. Both visits met the diagnostic criteria for probable IgG4-related ophthalmic disease [13]. In recent years, it has been suggested that IgG4-related disease can predispose patients to developing ocular adnexal malignant lymphoma [14]. Cheuk et al. reported 3 cases of lymphoma originating from IgG4-related lacrimal gland inflammation and found that 10% of malignant lymphomas in their study were evolved from IgG4-related disease [15]. A meta-analysis found that 2 cases of non-Hodgkin lymphoma (NHL) arose at the site of prior histologically confirmed IgG4-related disease: a diffuse large B cell lymphoma and a MALT lymphoma [16]. Some researchers believe that malignant lymphoma occurs in the setting of IgG4-related inflammation, whereas other reports indicate that lymphoma cells are capable of producing IgG4, either coexistent or not with benign IgG4-positive cells [17–19]. Moreover, serum IgG4 testing rate has increased significantly in the last 5 years (70%) compared to the previous 5 years (33%), reflecting the growing attention of IgG4-related disease which can provide a substrate for the emergence of lymphoma [1].

At the time of diagnosis of lymphoma, the immunohistological finding of the patient in our report was IgG4-negative, but serum IgG4 was elevated. We believe there are two reasons to explain this phenomenon. First, the patient received glucocorticoid therapy after surgery 1 year prior. The subsequent IgG4-negative result might be an indication of effective treatment. However, a transition from positive to negative IgG4+ cells in orbital tissue within 1 year did not mean that the overall disease had subsided. IgG4-related disease is an immune-mediated disease that can cause fibroinflammatory lesions in nearly any organ. Therefore, high serum IgG4 level might be due to disease in other organs. Second, it is often difficult to evaluate IgG4-positive versus IgG-positive cells due to the background staining, especially in very small samples. According to the 2020 revised comprehensive diagnostic criteria for IgG4-RD, poor IgG and/or IgG4 staining cannot completely exclude IgG4-RD [20].

The clinical and radiological features of this patient lack specificity. Five visits over 6 years showed prominent enlargement of the right inferior rectus muscle belly, which is similar to the presentation of thyroid-associated ophthalmopathy (TAO). However, by carefully analyzing the patient’s imaging and exam results, there were differences between the patient’s condition and TAO. First, the typical radiological finding of TAO is a fusiform enlargement of the muscle belly, whereas this patient’s muscle had an oval change. Second, in TAO, if the right inferior rectus muscle develops to such a severe level, the rest of the extraocular muscles are also likely to be heavily involved, which was not observed in this patient. Third, the thyroid function was normal in any of all five of the visits. Fourth, the biopsy found a friable mass with a grayish fish-like cut surface, which is more consistent with the gross characteristics of malignant tumors. Even in patients with a confirmed diagnosis of TAO, continued deterioration should raise a suspicion of malignancy. Zoran et al. reported a patient with unilateral, low-grade marginal zone B-cell lymphoma simulating unilaterally worsening TAO. His previous symptoms worsened with progressive unilateral exophthalmos resistant to the previously applied glucocorticoid therapy [21].

This case demonstrates an atypical manifestation and course of disease in ocular adnexal lymphoma. There are two implications of our findings. First, we found that the long-term extraocular myositis may evolve into lymphoma and elevated IgG4 levels may be a precursor to the disease. Therefore, clinicians should not underestimate the potential severity of chronic myositis and should treat it proactively. Second, the clinical and radiological presentations of isolated lymphoma involving extraocular muscles are similar to those of TAO. In patients with painless extraocular muscle hypertrophy who lack systemic features of hyperthyroidism or hypothyroidism, malignancy should be considered and diagnosis confirmation by biopsy is warranted if necessary.

## Data Availability

The data generated during the present study is available upon reasonable request from the corresponding author at jmma@sina.com.

## Abbreviations

MALT lymphoma: extranodal marginal zone B-cell lymphoma of mucosa-associated lymphoid tissue
TAO: thyroid-associated ophthalmopathy
CBC: complete blood count
EBV: Epstein–Barr virus
IgG4-RD: IgG4-related disease
IgG4-ROD: IgG4-related ophthalmic disease
MRI: Magnetic resonance imaging
H&E: Hematoxylin-eosin
NHL: Non-Hodgkin lymphoma
HPF: High power field
